# A Novel Protein Encoded by Exosomal CircATG4B Induces Oxaliplatin Resistance in Colorectal Cancer by Promoting Autophagy

**DOI:** 10.1002/advs.202204513

**Published:** 2022-10-26

**Authors:** Zihao Pan, Jun Zheng, Jiebin Zhang, Jiatong Lin, Jianguo Lai, Zejian Lyu, Huolun Feng, Junjiang Wang, Deqing Wu, Yong Li

**Affiliations:** ^1^ Department of Gastrointestinal Surgery Department of General Surgery Guangdong Provincial People's Hospital Guangdong Academy of Medical Sciences Guangzhou 510080 China; ^2^ The Second School of Clinical Medicine Southern Medical University Guangzhou 510080 China; ^3^ Department of Hepatic Surgery and Liver Transplantation Center of the Third Affiliated Hospital of Sun Yat‐sen University Organ Transplantation Research Center of Guangdong Province Guangdong Province Engineering Laboratory for Transplantation Medicine Guangzhou 510630 China; ^4^ School of medicine South China University of Technology Guangzhou Guangdong Province 510006 China; ^5^ Department of Breast Cancer Cancer Center Guangdong Provincial People's Hospital Guangdong Academy of Medical Sciences Guangzhou 510080 China

**Keywords:** autophagy, chemoresistance, circular RNA, colorectal cancer (CRC)

## Abstract

Oxaliplatin is commonly used in chemotherapeutic regimens for colorectal cancer (CRC) after surgical resection. However, acquired chemoresistance seriously affects the curative effect in CRC patients, and the mechanism is still unclear. Here, a circular RNA, circATG4B is identified, which plays an important role in oxaliplatin resistance in CRC. circATG4B expression is found to be increased in exosomes secreted by oxaliplatin‐resistant CRC cells. In addition, the results suggest that circATG4B induces oxaliplatin resistance by promoting autophagy. Further in vivo and in vitro studies indicate that the effect of circATG4B is attributed to its potential to encode a novel protein, circATG4B‐222aa. Next, circATG4B‐222aa is found to function as a decoy to competitively interact with TMED10 and prevent TMED10 from binding to ATG4B, which leads to increased autophagy followed by induction of chemoresistance. Therefore, this study reveals that exosomal circATG4B participates in the decreased chemosensitivity of CRC cells, providing a new rationale for a potential therapeutic target for oxaliplatin resistance in CRC.

## Graphical abstract

In recent years, significant attention has focused on circRNA translation to determine its clinical significance. The present study describes the physiological role of a novel protein encoded by circATG4B. These findings may shed light on the protein‐coding potential of circRNAs during drug resistance progression and provide a precise intervention target for the treatment of chemoresistant colorectal cancer.

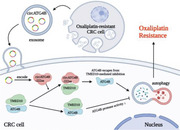

## 1 Introduction

Colorectal cancer (CRC) is one of the most common malignancies with an increasingly high incidence worldwide, and is the second leading cause of cancer‐related death globally.^[^ ^1^ ^]^ Although the clinical outcomes of CRC patients have been improved by the development of therapeutic approaches, the overall survival of CRC patients is still unsatisfactory. Oxaliplatin (L‐OHP), a third‐generation platinum compound that inhibits gene transcription and DNA synthesis, is commonly used in chemotherapeutic regimens after surgical resection to treat CRC patients.^[^ ^2^ ^]^ However, cancer cells eventually develop chemoresistance, a phenomenon that has historically impeded the effectiveness of oxaliplatin via various mechanisms, including decreased drug uptake, enhanced drug deactivation, enhanced DNA repair, activated cell proliferation or suppressed cell death. Therefore, it is essential to delineate the precise molecular mechanisms by which CRC cells develop resistance to oxaliplatin, identify effective biomarkers to analyze the oxaliplatin response, and develop targeted treatments to minimize resistance.

There is increasing evidence that exosomes, 100–300 nm diameter vesicles, secreted by cancer cells can transfer oncogenic molecules,^[^ ^3^, ^4^ ^]^ including noncoding RNAs (ncRNAs) or proteins, to the targeted cells involved in tumor invasion, proliferation immune escape and drug resistance. In addition, circular RNAs (circRNAs) are endogenous ncRNAs that are characterized by the connection of a 5′ splice site to the 3′ splice site of an upstream exon or intron.^[^ ^5^ ^]^ Acting as miRNA sponge is the most commonly reported function of circRNAs.^[^ ^6^, ^7^ ^]^ Additionally, circRNAs can bind to target proteins and regulate their biological behaviors.^[^ ^8^ ^]^ Unlike linear RNAs, circRNAs were previously considered to be untranslatable because they lack the traditional essential elements for translation, such as a 5′ cap and a poly(A) tail.^[^ ^9^ ^]^ However, several recent studies have reported that circRNAs can actually encode proteins.^[^ ^10^, ^11^ ^]^ Moreover, circRNAs enriched in exosomes are important for intercellular communication.^[^ ^12^, ^13^ ^]^ Previous studies have shown that exosomal circRNAs play vital roles in mediating biological behaviors.^[^ ^14^, ^15^ ^]^ As exosomes enrich a variety of circRNAs, the functions and underlying mechanisms of cancer‐derived exosomal circRNAs in CRC chemoresistance require further investigation.

Autophagy is a conserved biological process through which cellular components and damaged organelles are sequestered in autophagosomes for lysosomal degradation, and it is accepted that it is a key mechanism in promoting cancer cell survival and chemoresistance.^[^ ^16^, ^17^, ^18^, ^19^, ^20^ ^]^ A key autophagy‐related factor, ATG4B cleaves MAP1LC3/LC3 to generate LC3‐I and is subsequently conjugated to phosphatidylethanolamine (PE), forming lipidated LC3‐II on phagophore membranes.^[^ ^21^, ^22^ ^]^ In addition, ATG4B expression has been demonstrated to be positively correlated with cancer chemoresistance. For example, Lei et al. showed that upregulation of ATG4B can induce autophagy followed by suppression of cisplatin (DDP) chemosensitivity in gastric cancer cells.^[^ ^23^ ^]^ In CRC, previous studies have also revealed the effects of ATG4B on enhancing cancer cell chemoresistance to oxaliplatin and 5‐fluorouracil (5‐FU).^[^ ^24^, ^25^ ^]^ However, whether the circRNA derived from ATG4B is associated with the process of autophagy to induce chemoresistance in CRC is unclear.

In this study, we found that the level of a novel circRNA (circATG4B, circ_0 007159) derived from ATG4B was increased in exosomes secreted by oxaliplatin‐resistant CRC cells. We found that exosomal circATG4B induced autophagy and oxaliplatin resistance by encoding a novel protein (circATG4B‐222aa) that interacts with TMED10 (a member of the 24 cargo receptor family that is involved in vesicular protein trafficking in the early secretory pathway^[^ ^26^ ^]^). Therefore, this study revealed that exosomal circATG4B could decrease the chemosensitivity of CRC cells, providing a new rationale for a potential therapeutic target for oxaliplatin resistance in CRC.

## 2 Results

### 2.1 Analysis of ATG4B‐Related CircRNAs in Oxaliplatin‐Resistant CRC

**Figure 1:**
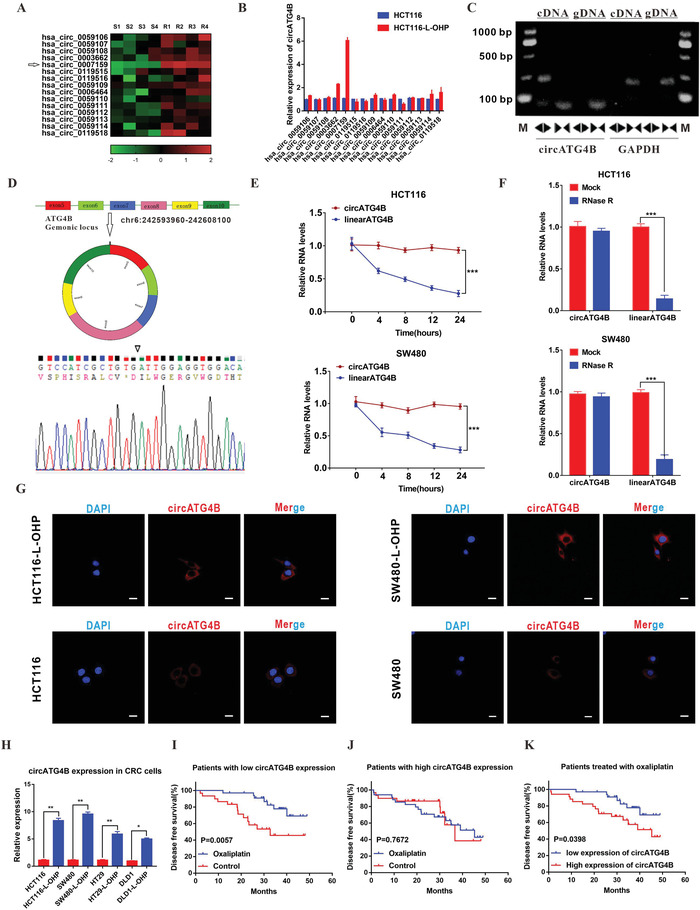
CircATG4B is overexpressed in oxaliplatin‐resistant CRC cells. A) Heatmap showing ATG4B gene‐derived circRNAs in CRC chemoresistant tissues compared with parental tissues analyzed by qRT‐PCR. B) The expression of circRNAs in HCT116 and HCT116‐L‐OHP (chemoresistant) cells. C) Divergent primer detected circular RNAs in cDNA but not gDNA. D) Six exons form circATG4B by back splicing from chromosomal region and Sanger sequencing of circATG4B showed the back‐splice junction (∇). E) Relative RNA level of circATG4B and ATG4B mRNA in different time points. F) Relative RNA level of circATG4B and ATG4B mRNA treated with RNase R. G) Fluorescence in situ hybridization assay was conducted to determine the subcellular localization of circATG4B in chemoresistant and parental CRC cells. Scale bars, 20 µm. H) The circATG4B expression of chemoresistant CRC cells was significantly higher than the parental CRC cells. I) Kaplan–Meier survival curves analysis of DFS in low‐circATG4B‐expression CRC patients with or without oxaliplatin therapy (*n* = 64). J) Kaplan–Meier survival curves analysis of DFS in high‐circATG4B‐expression CRC patients with or without oxaliplatin therapy (*n* = 64). K) Kaplan–Meier survival curves analysis of DFS in different circATG4B‐expression CRC patients with oxaliplatin therapy. The median value of circATG4B expression level was used as a cut‐off. *p < 0.05, **p < 0.01, ***p < 0.001.

ATG4B has been reported to be associated with chemoresistance due to its autophagy‐inducing function in various cancers.^[^ ^27^, ^28^, ^29^ ^]^ Additionally, increasing evidence indicates that circRNAs are produced by the back‐splicing of precursor mRNAs, and some circRNAs are involved in their host gene functions. Therefore, the expression of 15 circRNAs originating from ATG4B was evaluated (we obtained circRNA sequencing data from the online database circBase). We found that hsa_circ_0 007159 (circATG4B) was obviously upregulated in CRC tissues of oxaliplatin‐resistant patients (**Figure** **1**A), which was consistent with the status of cell expression (HCT116‐L‐OHP, chemoresistant CRC cells) (Figure 1B). To further validate the existence of circATG4B, the PCR products were assessed using two sets of primers. The linear form of ATG4B was only amplified by the convergent primers, while the circular form was only amplified by the divergent primers. The PCR products were validated by electrophoresis using cDNA and genomic DNA (gDNA) as templates, and the results showed that the product around the expected size was only observed in cDNA amplified by divergent primers, but not in genomic DNA (gDNA) (Figure 1C). In addition, Sanger sequencing confirmed the head‐to‐tail splicing of the amplified circATG4B, suggesting that circATG4B was derived from the exons of the ATG4B gene, consistent with the circATG4B data from circBase (Figure 1D). Compared with the linear form, circATG4B has a longer half‐life and is resistant to RNase R treatment (Figure 1E,F), further suggesting that circATG4B indeed exists in a circular form. The results from fluorescence in situ hybridization (FISH) showed that circATG4B was mainly localized in the cytoplasm and much more abundant in oxaliplatin‐resistant CRC cells (HCT116‐L‐OHP, SW480‐L‐OHP) than in their sensitive counterparts (Figure 1G). Next, we assessed the circATG4B level in several oxaliplatin‐resistant cell lines and the matched CRC cells. Consistent with the above observations, we found that circATG4B was significantly increased in oxaliplatin‐resistant CRC cells (Figure 1H). Additionally, we examined circATG4B expression in 128 CRC patient tissues and classified patients into low‐ and high‐level groups according to the median circATG4B expression. The baseline clinical characteristics of these patients are listed in Table S4, Supporting Information. Kaplan–Meier survival analysis indicated that among patients with low circATG4B expression, the patients receiving oxaliplatin treatment had significantly longer disease‐free survival (DFS) times than those in the control group (*p* = 0.0057) (Figure 1I). Conversely, no difference was found in the DFS time between the oxaliplatin treatment group and the control group among patients with high circATG4B levels (Figure 1J). Moreover, we found that oxaliplatin treatment was an independent prognostic factor (oxaliplatin treatment versus no treatment, HR = 0.320, 95% CI 0.124‐0.824, *p* = 0.018) only in patients with low circATG4B expression and not in patients with high circATG4B expression (**Table** **1**). Moreover, patients with higher circATG4B expression had worse DFS compared with those with low circATG4B expression in the cohort treated with oxaliplatin (*p* = 0.0398) (Figure 1K). Taken together, these results indicated that circATG4B was closely correlated with the oxaliplatin response in CRC patients.

**Table 1:** Univariate and multivariate Cox‐regression analysis of prognostic factors for CRC patients

	Low expression			High expression		
	HR	95% CI	P	HR	95% CI	P
Univariate analysis						
TNM stage						
I and II	1			1		
III and IV	1.038	(0.347, 3.108)	0.947	1.025	(0.344, 3.035)	0.965
Tumor location						
Colon	1			1		
Rectum	1.693	(0.795, 4.664)	0.146	0.997	(0.430, 2.314)	0.995
Differentiation						
Well/ Moderate	1			1		
Poor	1.419	(0.472, 4.265)	0.534	1.162	(0.395, 3.422)	0.785
Chemotherapy						
Control	1			1		
Oxaliplatin	0.291	(0.115,0.736)	0.009^a)^	1.142	(0.474, 2.752)	0.767
Age	1.047	(1.004,1.091)	0.031^a)^	1.005	(0.974, 1.0369)	0.733
Multivariate analysis						
Chemotherapy						
Control	1					
Oxaliplatin	0.320	(0.124,0.824)	0.018^a)^			
Age	1.039	(0.996,1.083)	0.073			

^a)^ *p* < 0.05.

### 2.2 CircATG4B Transferred via Exosomes Induces Oxaliplatin Resistance

**Figure 2:**
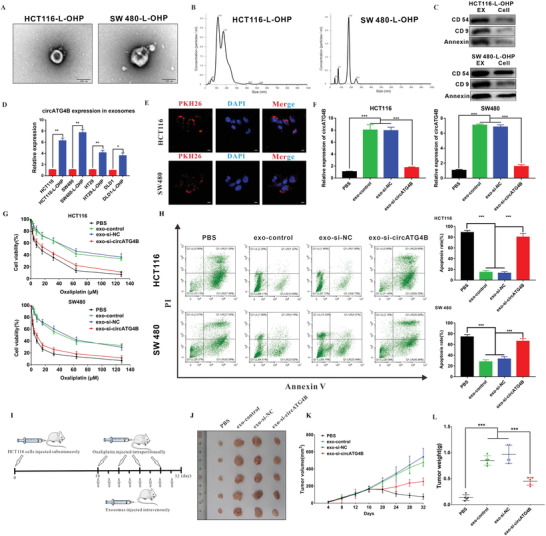
CircATG4B transferred by an exosomal manner induces oxaliplatin resistance. A) Exosomes were isolated from the supernatant of the culture medium of HCT116‐L‐OHP and SW480‐L‐OHP cells, and the morphology and size were confirmed by transmission electron microscopy. Scale bars, 100 nm. B) NTA distribution of CRC cell‐derived exosomes. C) CRC cell‐derived exosomes were analyzed by Western blotting using anti‐CD54, anti‐CD9, and anti‐Annexin antibodies. Cellular lysates were used as positive loading controls. D) The circATG4B expression of the chemoresistant‐CRC exosome was significantly higher than the exosome of parental CRC cells. E) PKH26‐labeled CRC‐L‐OHP exosomes could fuse into CRC cells. Scale bars, 10 µm. F) qRT‐PCR quantification of circATG4B with the treatment of diverse CRC‐L‐OHP exosomes in CRC cells. G) CCK‐8 detection of cell viability by oxaliplatin in CRC cells treated with diverse CRC‐L‐OHP exosomes. H) Flow cytometry analysis of cell apoptosis by oxaliplatin in CRC cells treated with diverse CRC‐L‐OHP exosomes. I) A flow chart depicting the in vivo experimental design. J) The tumor of different groups treated with oxaliplatin. K) Tumor grow rates are showed in different groups. L) Tumor weights were monitored in different groups. *p < 0.05, **p < 0.01, ***p < 0.001.

Previous studies have demonstrated that circRNA can be packaged into exosomes and transferred from cell to cell.^[^ ^30^ ^]^ Therefore, we hypothesized that circATG4B might be transferred from oxaliplatin‐resistant CRC cells to oxaliplatin‐sensitive cells through exosomes, a targeted delivery approach. To verify this hypothesis, exosomes isolated from the supernatant of oxaliplatin‐resistant CRC cells were identified via electron microscopy by their typical structures as round particles with a bilayer membrane (**Figure** **2**A). Furthermore, the size distribution of the exosomes was analyzed by nanoparticle tracking analysis (NTA) (Figure 2B). Additionally, Western blotting revealed the expression of exosomal markers, including CD54, CD9 and Annexin (Figure 2C). Then, real‐time quantitative polymerase chain reaction (qRT‐PCR) analyses of exosomes were conducted in four different CRC oxaliplatin‐resistant cell lines and paired CRC cells. The results showed that circATG4B was markedly upregulated in the oxaliplatin‐resistant CRC cell lines, especially in the exosomes derived from the HCT116‐L‐OHP and SW480‐L‐OHP cell lines (Figure 2D), which was consistent with the cell expression levels. When CRC cells were cocultured with exosomes (labelled with PKH26) derived from oxaliplatin‐resistant CRC cells, we observed a large amount of exosomes (red) in the recipient cells, indicating the high exosome uptake efficiency of CRC cells (Figure 2E). qRT‐PCR also showed that exosomes isolated from the culture medium of chemoresistant CRC cells contained more circATG4B than those isolated from the culture medium of parental cells (Figure S1A, Supporting Information). Moreover, circATG4B was more enriched in exosomes isolated from the culture medium of oxaliplatin‐resistant cells than in the cytosol (Figure S1B, Supporting Information), further suggesting that circATG4B in oxaliplatin‐resistant CRC cells can be transferred via exosomes. Furthermore, we knocked down exosomal circATG4B by siRNA in oxaliplatin‐resistant CRC cells, and the exosomes were subsequently isolated (Figures S1C and S1D, Supporting Information). After incubation with the isolated exosomes or PBS in the parental cells, we found that circATG4B was significantly upregulated due to coculture with exosomes from oxaliplatin‐resistant cells treated with or without si‐NC (negative control), and this phenomenon was reversed when exosomal circATG4B was knocked down by siRNA (Figure 2F).

Given that exosomal circATG4B was derived from oxaliplatin‐resistant cells, we further investigated its effect on chemoresistance. Our results showed that compared to the incubation of exo‐si‐NC, incubation of exo‐si‐circATG4B increased the inhibition rate and induction of apoptosis upon oxaliplatin treatment in CRC cells (Figure 2G,H). Colony formation assays indicated that lower oxaliplatin resistance was observed in parental CRC cells after treatment with exo‐si‐circATG4B than after treatment with exo‐si‐NC (Figure S1E, Supporting Information). To explore the effects of exosomal circATG4B in vivo, a xenograft mouse model was established by subcutaneous injection of HCT116 cells (*n* = 5 for each group). Sixteen days after injection, various exosomes or an equivalent volume of PBS was injected intravenously every 2 days, and oxaliplatin was injected intraperitoneally every 4 days. The size of tumors was measured every 4 days, and all mice were sacrificed after 16 days (Figure 2I). The growth rates and tumors weights of the tumors treated with exo‐si‐NC were higher than those of tumors treated with exo‐si‐circATG4B or PBS (Figure 2J–L), indicating that exosomal circATG4B decreased sensitivity to oxaliplatin in CRC.

### 2.3 Exosomal CircATG4B Enhances Autophagic Activity in the Oxaliplatin Resistance Process

**Figure 3:**
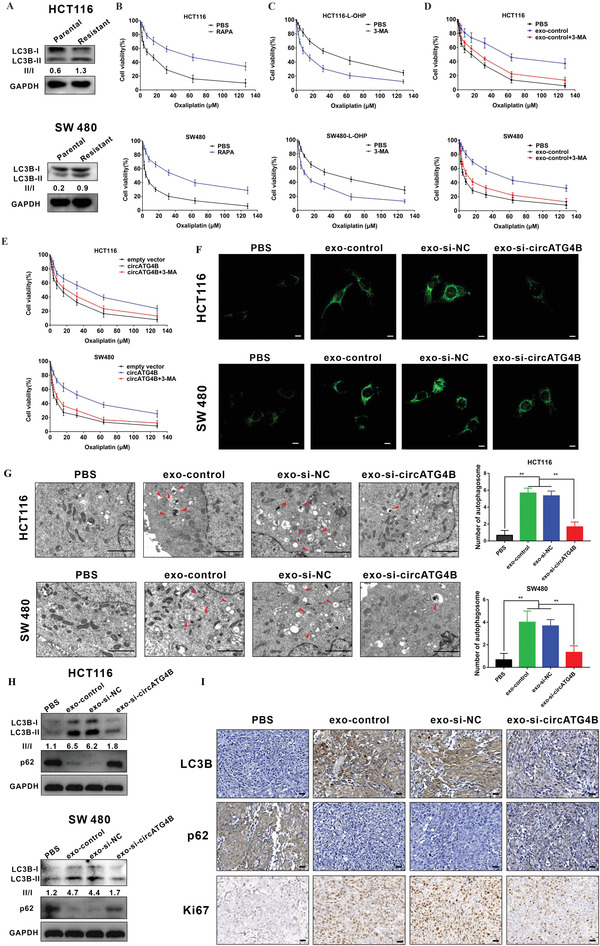
Exosomal circATG4B promotes autophagy level. A) Western blotting showed that oxaliplatin‐resistant CRC cells induced the increased proportion of LC3B‐II/LC3B‐I. B,C) RAPA increased oxaliplatin resistance of CRC cells (B) while 3‐MA attenuated in chemoresistance of oxaliplatin‐resistant cells (C). D) 3‐MA reversed the chemoresistance induced by exosome‐control in sensitive cells. E) 3‐MA abolished circATG4B‐promoted oxaliplatin resistance in sensitive cells. F) Puncta‐like staining detection of CRC cells transfected with various treatments is shown. Scale bars, 10 µm. G) Transmission electron microscopy analysis of autophagy is shown. Arrows: autophagosomes/autolysosomes. Scale bars, 2 µm. H) LC3B‐II accumulation and p62 of CRC cells transfected with various treatments is shown. I) IHC analysis of Ki67, LC3B, and p62 in subcutaneous tumor. Scale bars, 20 µm.

Autophagy plays an important role in chemoresistance processes, according to previous studies. Therefore, we measured the autophagy level in oxaliplatin‐resistant cells and parental cells. Remarkably, a higher ratio of LC3B‐II/I was observed in oxaliplatin‐resistant cells than in the corresponding parental cells (**Figure** **3**A). Additionally, we also observed this phenomenon in clinical samples by immunohistochemistry (IHC) staining (Figure S2, Supporting Information), further suggesting that the autophagy level is increased upon acquisition of oxaliplatin resistance. To identify whether autophagy is essential for oxaliplatin resistance, we treated CRC cells with rapamycin (RAPA; 3 mmol L^−1^ for 48 h), a common autophagy inducer. We found that RAPA treatment induced chemoresistance (Figure 3B). Conversely, we also found that 3‐methyladenine (3‐MA; 10 mmol L^−1^ for 48 h), a common autophagy inhibitor, increased oxaliplatin sensitivity in resistant CRC cells (Figure 3C), further suggesting that autophagy plays an important role in oxaliplatin resistance. Furthermore, we found that CRC cells treated with exo‐control showed higher drug resistance than cells treated with PBS and that this chemoresistance was greatly reversed by treatment with 3‐MA (Figure 3D), indicating that exosomes might facilitate the switch from drug‐sensitivity to drug‐resistance by regulating autophagy. Given that exosomal circATG4B can induce oxaliplatin resistance and is produced by the back‐splicing of an autophagy‐related gene (ATG4B), we speculated that circATG4B is related to oxaliplatin resistance and autophagy. The results indicated that chemoresistance was increased in CRC cells transfected with the circATG4B overexpression plasmid. However, treatment with 3‐MA reversed this effect (Figure 3E), suggesting that circATG4B could induce chemoresistance through autophagy. Moreover, we explored the role of exosomal circATG4B in the autophagy process. After treatment with diverse exosomes secreted by oxaliplatin‐resistant CRC cells, a series of experiments were performed in the recipient cell lines HCT116 and SW480. As shown by the results of immunofluorescence (IF) staining, CRC cells pretreated with exo‐si‐NC showed a significant increase in autophagosome formation, whereas CRC cells pretreated with exosomes with a reduced level of circATG4B showed a decrease in the number of green puncta (Figure 3F). Furthermore, transmission electron microscopy (TEM) analysis showed that the number of autophagosomes was increased after pretreatment with exo‐si‐NC but significantly decreased after pretreatment with exo‐si‐circATG4B (Figure 3G). Additionally, LC3B‐I to II conversion was markedly promoted by treatment with exosomes secreted by oxaliplatin‐resistant CRC cells transfected with si‐NC compared to treatment with PBS, while LC3B‐II accumulation was suppressed when exosomal circATG4B was knocked down. (Figure 3H). Moreover, the IHC results in the previously established xenograft mouse model showed that the expression level of p62 was significantly increased but those of LC3B and Ki67 (a marker of cell proliferation^[^ ^31^ ^]^) were decreased in CRC cells treated with exo‐si‐circATG4B compared with the exo‐si‐NC group (Figure 3I), which further confirmed that exosomal circATG4B induced oxaliplatin resistance by regulating autophagy.

### 2.4 CircATG4B Encodes a Novel 222 Amino Acid (aa) Protein, circATG4B‐222aa

**Figure 4:**
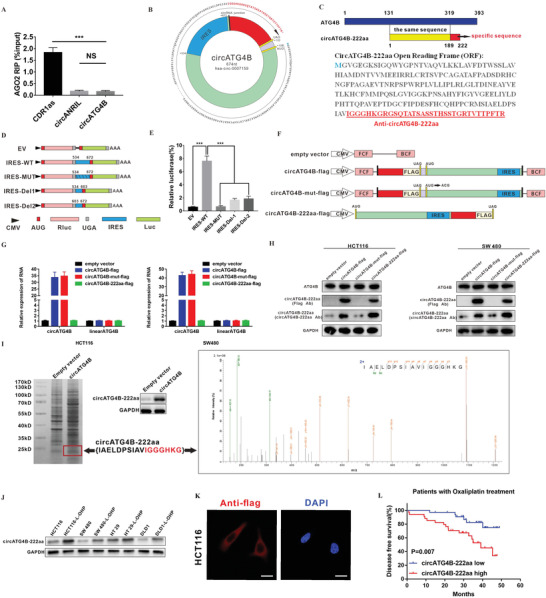
Evaluation of the coding ability of circATG4B. A) RIP assay indicated that circATG4B was not associated with AGO2. B) The putative open reading frame (ORF) in circATG4B. C) Upper panel, circATG4B‐222aa shares the majority of its amino acids sequence with that of ATG4B with the exception of the unique amino acids. Lower panel, the sequences of putative ORF are shown. D) The putative IRES activity of circATG4B was tested. IRES sequences in circATG4B or its different truncation/mutation are cloned between the Rluc and Luc reporter genes with independent start (AUG) and stop (UGA) codons. E) The relative luciferase activity of Luc/Rluc in the above vectors was tested. F) Four vectors were constructed. Empty vector; circATG4B‐flag: flag‐tagged circATG4B sequence cloned into a CMV‐induced expression vector; circATG4B‐mut‐flag: flag‐tagged circATG4B sequence with a mutated start codon (ATG→ACG) cloned into a CMV‐induced expression vector; circATG4B‐222aa‐flag: flag‐tagged circATG4B‐222aa sequence cloned into a CMV‐induced expression vector. Forward circRNA frame (FCF) and backward circRNA frame (BCF) are sequences which could circularize the sequence of circRNA. G) Relative RNA expression of circATG4B and linearATG4B was detected by qRT‐PCR. H) Flag antibody and circATG4B‐222aa antibody were used to detect circATG4B‐222aa expression in CRC cells transfected with the above vectors. I) Left panel, total proteins from circATG4B or control plasmid‐transfected HEK‐293T cells were separated via SDS‐PAGE. CircATG4B‐222aa overexpression was confirmed by immunoblotting. Right panel, circATG4B‐222aa junction‐specific peptides were identified by LC/MS. J) CircATG4B‐222aa in chemoresistant and parental CRC cells was detected by immunoblotting. K) Flag‐tagged circATG4B was transfected into HCT116 cells. Immunofluorescence staining by anti‐flag was performed to show the circATG4B‐222aa. Scale bars, 20 µm. L) Semi‐quantitative analysis of circATG4B‐222aa expression level and CRC patient disease‐free survival (DFS) in the 68 patient cohort treated with Oxaliplatin. ***p < 0.001.

MicroRNA (miRNA) sponging is the most common function of circRNAs, and AGO2 is a key component of the miRNA‐containing RISC complex, which is essential for the sequestration of miRNAs by circRNAs.^[^ ^8^ ^]^ However, the RIP results revealed that circATG4B was not associated with AGO2 (**Figure** **4**A), suggesting that circATG4B might perform its functions via a different mechanism. Increasing evidence has shown that some circRNAs are able to encode novel proteins. In addition, we found that there was a 669‐nt open reading frame (ORF) in circATG4B, which had the potential to encode a 222 amino‐acid (aa) protein, through consulting the annotations in circRNA Db (Figure 4B; Figure S3, Supporting Information). In the ORF of circATG4B, the tandem “AUG” codons within the circRNA sequence could initiate the translation of a novel protein, while the termination codon “UAG” could terminate translation. The novel protein, termed “circATG4B‐222aa”, contained a new sequence with 33 specific amino acids (Figure 4C). Because circRNAs have no 5′‐cap sequence, which can initiate protein translation, an internal ribosomal entry site (IRES) is required. Furthermore, we found an IRES in the ORF (from +534 to +672), and the activity of the IRES in circATG4B was verified by using a dual‐luciferase assay (Figure 4D,E). To confirm the existence of circATG4B‐222aa, we constructed four different flag‐tagged vectors (empty vector; circATG4B‐flag: flag‐tagged circATG4B sequence cloned into a CMV‐induced expression vector; circATG4B‐mut‐flag: flag‐tagged circATG4B sequence with a mutated start codon (ATG→ACG) cloned into a CMV‐induced expression vector; circATG4B‐222aa‐flag: flag‐tagged circATG4B‐222aa sequence cloned into a CMV‐induced expression vector) (Figure 4F). In CRC cells transfected with the circATG4B‐flag vector or the circATG4B‐flag‐mut vector, the expression of circATG4B was significantly upregulated. Compared to transfection with empty vector, transfection with the circATG4B‐222aa vector did not obviously change the expression of circATG4B. None of these four vectors affected the level of linear ATG4B mRNA (Figure 4G). Furthermore, flag‐tagged circATG4B‐222aa protein was detected using anti‐flag antibodies at ≈24 kDa after circATG4B‐flag or circATG4B‐222aa‐flag transfection. Additionally, we developed a novel antibody that was able to specifically recognize the unique circATG4B‐222aa sequence. Using the circATG4B‐222aa‐specific antibody, circATG4B‐222aa was detected in CRC cells transfected with empty vectors and circATG4B‐flag‐mut, while circATG4B‐flag‐ or circATG4B‐222aa‐flag‐transfected cells showed higher expression of circATG4B‐222aa, suggesting that circATG4B‐222aa was endogenously expressed. Moreover, ATG4B expression was not affected by any of the four vectors (Figure 4H). Finally, total protein from circATG4B‐ and empty vector‐transfected HEK‐293T cells were separated via SDS‐PAGE, and protein bands with a size near 24 kD were subsequently excised and submitted for LC‐MS/MS. The unique peptide sequence (IAELDPSIAVIGGGHKG) formed by the splicing junction of circATG4B was successfully identified, indicating that circATG4B could encode a novel protein (Figure 4I). Additionally, the expression of circATG4B‐222aa was obviously higher in chemoresistant CRC cell lines (Figure 4J). Immunofluorescence staining was performed using an anti‐flag antibody to identify the cytoplasmic localization of circATG4B‐222aa after we transfected flag‐tagged circATG4B‐222aa into HCT116 cells (Figure 4K). Finally, circATG4B‐222aa expression was positively correlated with poor prognosis in CRC patients treated with oxaliplatin treatment, indicating that circATG4B‐222aa may be a prognostic marker for oxaliplatin resistance (Figure 4L).

### 2.5 CircATG4B‐222aa but not CircATG4B Increases Autophagy and Induces Oxaliplatin Resistance in CRC Cells In Vitro and In Vivo

**Figure 5:**
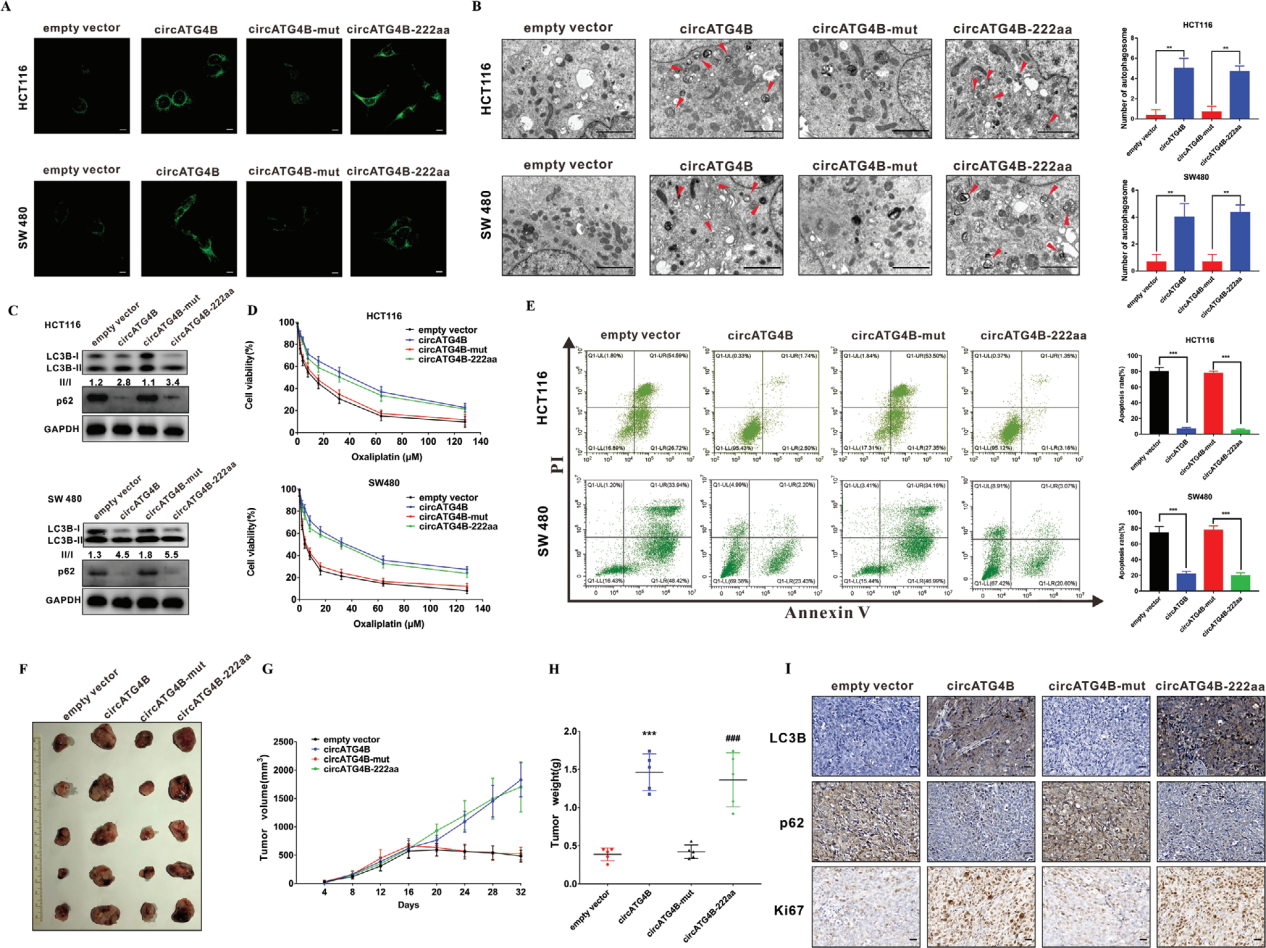
CircATG4B‐222aa but not circATG4B increases the autophagy and induces oxaliplatin in CRC cells in vitro and in vivo. A) Puncta‐like staining detection of HCT116 and SW480 transfected with four vectors. Scale bars, 10 µm. B) Transmission electron microscopy analysis of autophagy of HCT116 and SW480 transfected with four vectors. Scale bars, 2 µm. C) LC3B‐II/LC3B‐I proportion of HCT116 and SW480 transfected with four vectors. D) CCK‐8 detection of cell viability by oxaliplatin in HCT116 and SW480 transfected with four vectors. E) Flow cytometry analysis of cell apoptosis by oxaliplatin in HCT116 and SW480 transfected with four vectors. F) Nude mice xenografts were formed by HCT116 cells transfected with empty‐vector, circATG4B, circATG4B‐mut, and circATG4B‐222aa, respectively. Then, sixteen days after injection, oxaliplatin was injected intraperitoneally. G) Tumor volumes were monitored during the time course. H) Tumor weights were also measured at the end of this study. I) IHC analysis of LC3B, p62, and Ki67 in tumor nodules. Scale bars, 20 µm. **p < 0.01, ***p < 0.001; ***p < 0.001 empty vector versus circATG4B group, ### *p* < 0.001 circATG4B‐mut versus circATG4B‐222aa group (H).

To explore the biological function of circATG4B‐222aa, the above four vectors without the flag tag were transfected into CRC cell lines. The results indicated that the number of LC3B puncta, autophagosome formation and LC3B‐I/II conversion were significantly increased by transfection with the circATG4B or circATG4B‐222aa overexpression vectors, as shown by fluorescence microscopy, TEM and Western blotting (**Figure** **5**A–C). However, there was no obvious change in the CRC cells transfected with the circATG4B‐mut vector, which was predicted to be unable to initiate the translation of circATG4B‐222aa. These results suggested that overexpression of only circATG4B‐222aa increased the level of autophagy.

Intriguingly, overexpression of circATG4B‐222aa decreased the oxaliplatin sensitivity and oxaliplatin‐induced apoptosis. However, overexpression of mutant circATG4B did not obviously influence these phenomena (Figure 5D,E). Colony formation assays similarly revealed that CRC parental cells acquired oxaliplatin resistance after circATG4B and circATG4B‐222aa overexpression (Figure S4, Supporting Information). To further investigate the effect of circATG4B‐222aa in vivo, xenograft mouse models were established by subcutaneous injection of HCT116 cells stably transfected with different circATG4B vectors. Then, sixteen days after injection, oxaliplatin was injected intraperitoneally every 4 days. The circATG4B and circATG4B‐222aa groups had larger growth rates and tumor weights than the vector control and circATG4B‐mut groups (Figure 5F–H). We assessed the expression of LC3B, p62, and Ki67 in the tumors of nude mice. The IHC results indicated that the expression levels of LC3B and Ki67 were significantly increased but that of p62 was decreased in the circATG4B and circATG4B‐222aa groups compared with the control and circATG4B‐mut groups (Figure 5I). In summary, these results suggested that the encoded protein (circATG4B‐222aa) but not the circRNA (circATG4B), increases autophagy and induces oxaliplatin resistance in CRC cells.

### 2.6 CircATG4B‐222aa Protects against the Effect of ATG4B Induced by TMED10‐Mediated Inhibition

**Figure 6:**
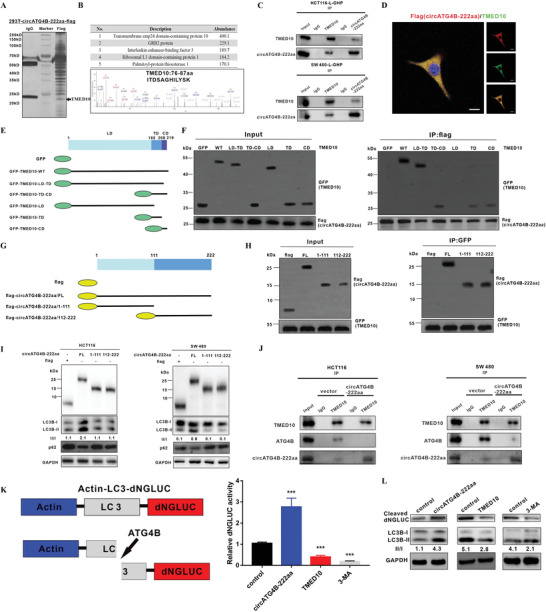
CircATG4B‐222aa protects against the effect of ATG4B induced by TMED10‐mediated inhibition. A) Total proteins from flag‐circATG4B‐222aa plasmid‐transfected HEK‐293T cells were extracted. The proteins coimmunoprecipitated with antibody against flag were separated via SDS‐PAGE. B) Upper panel, list of the top five differentially expressed proteins identified by mass spectrometry. Lower panel, TMED10 was identified by LC/LC‐MS. C) The interaction of TMED10 and circATG4B‐222aa was detected by immunoprecipitation in CRC cells. D) Flag‐tagged circATG4B‐222aa was transfected into HCT116 cells and immunofluorescence was performed using anti‐flag and anti‐TMED10 antibody. Scale bars, 10 µm. E) Schematic of domain structure of TMED10 and GFP‐tagged TMED10 mutants. F) HEK‐293T cells were transfected with flag‐tagged circATG4B‐222aa and GFP‐tagged full‐length or TMED10 fragments, followed by IP using anti‐flag antibody. G) Schematic diagrams showed the wild‐type circATG4B‐222aa and its truncation mutants. H) HEK‐293T cells were transfected with GFP‐tagged TMED10 and flag‐tagged circATG4B‐222aa mutants, followed by IP with anti‐GFP antibody. I) Western Blotting assays showed the levels of autophagy in cells after transfection of wild‐type circATG4B‐222aa ORF or truncation mutants. J) After overexpression of circATG4B‐222aa, the binding capacity between TMED10 and ATG4B was monitored by Co‐IP. K) Left panel, schematic diagram of the quantification of ATG4B activity using an assay based on a luciferase‐release system. Right panel, HCT116 cells transfected with pEAK12‐Actin‐LC3‐dNGLUC were transfected with control, or circATG4B‐222aa overexpression or TMED10 overexpression, or treated with 3‐MA. The supernatants were collected, and the relative luciferase activity was measured. L) The collected supernatants were analyzed by Western blotting using an anti‐luciferase and anti‐LC3 antibody.

To further explore the underlying mechanism of circATG4B‐222aa in autophagy, a Co‐IP assay and mass spectrometry were performed to investigate potential circATG4B‐222aa‐interacting proteins after transfecting the flag‐tagged circATG4B‐222aa plasmid into HEK‐293T cells. We identified the differentially expressed proteins (**Figure** **6**A) and found that the protein with the highest abundance in the ranking list of recognized proteins was TMED10 (Figure 6B). Therefore, we speculated that TMED10 is the potential target of circATG4B‐222aa. Furthermore, circATG4B‐222aa indeed interacted with TMED10 in CRC cells, as expected, which was confirmed by further experiments (Figure 6C). Immunofluorescence staining in HCT116 cells transfected with flag‐tagged circATG4B‐222aa also indicated the colocalization of circATG4B‐222aa with TMED10 (Figure 6D).

To explore the domains of TMED10 responsible for its interaction with circATG4B‐222aa, a series of GFP‐tagged TMED10 deletion mutants were generated (Figure 6E). Subsequent Co‐IP experiments combined with Western blotting demonstrated that compared with full‐length TMED10, C‐terminal deletion mutants containing the luminal domain‐transmembrane domain (LD‐TD), transmembrane domain‐cytosolic domain (TD‐CD), transmembrane domain (TD) and cytosolic domain (CD) interacted weakly with circATG4B‐222aa. However, the LD‐only mutant of TMED10 did not bind to circATG4B‐222aa, suggesting that the both TD and CD of TMED10 are required for the interaction between TMED10 and circATG4B‐222aa (Figure 6F). By reciprocal Co‐IP analysis with flag‐tagged truncation mutants of circATG4B‐222aa, we also identified the regions of circATG4B‐222aa involved in the TMED10‐circATG4B‐222aa interaction (Figure 6G). Both truncations of circATG4B‐222aa (aa 1–111) and circATG4B‐222aa (aa 112–222) could be pulled‐down by TMED10 (Figure 6H), indicating that full‐length circATG4B‐222aa interacted with TMED10.

A previous study demonstrated that TMED10 directly interacts with ATG4B and suppresses the effect of ATG4B on LC3 cleavage and indicated that downregulation of TMED10 might enhance the proteolytic activity of ATG4B.^[^ ^32^ ^]^ We also obtained similar result (Figures S5 and S6, Supporting Information). In further studies, the results showed that LC3B‐I/II conversion was increased by expression of circATG4B‐222aa but not by either of the truncation mutants, indicating that full‐length circATG4B‐222aa is responsible for the autophagy process (Figure 6I). Since circATG4B‐222aa shares the majority of its amino acid sequence with ATG4B, with the exception of the unique amino acids, we hypothesized that circATG4B‐222aa may compete with ATG4B for binding to TMED10.

Interestingly, the results of Co‐IP indicated that overexpression of circATG4B‐222aa impaired the interaction between TMED10 and ATG4B (Figure 6J), suggesting that circATG4B‐222aa caused dissociation of ATG4B from TMED10. A previous study also showed that dissociation of ATG4B from TMED10 resulted in increased ATG4B activity during autophagy.^[^ ^32^ ^]^ We further performed assays to confirm the effect of the interaction between TMED10 and circATG4B‐222aa in CRC cells. In brief, the proteolytic activity of ATG4B can be monitored by measuring Gaussia luciferase activity. CRC cells with overexpression of pEAK12‐Actin‐LC3‐dNGLUC, which is secreted after cleavage by ATG4B, were generated. Then, autophagy activation induced in these cells with different treatments was measured by a luciferase activity assay. Luciferase activity was significantly decreased in TMED10‐transfected cells and 3‐MA‐treated cells compared to control cells. However, luciferase activity was increased by circATG4B‐222aa, suggesting that increasing the circATG4B‐222aa level enhances the cleavage of GLUC‐linked LC3 by activating ATG4B in CRC cells (Figure 6K). Furthermore, cleavage of Actin‐LC3‐dNGLUC in these cells was assessed using Western blotting. The release of luciferase fragments upon cleavage by ATG4B and LC3B‐I/II conversion was also significantly decreased in TMED10‐upregulated cells, while circATG4B‐222aa upregulation had the opposite effect (Figure 6L). Taken together, these findings indicated that circATG4B‐222aa might function as a decoy for TMED10, leading to escape from TMED10‐induced ATG4B inhibition.

### 2.7 CircATG4B‐222aa Reverses the Chemosensitivity Induced by TMED10 in Oxaliplatin‐Resistant CRC Cells

**Figure 7:**
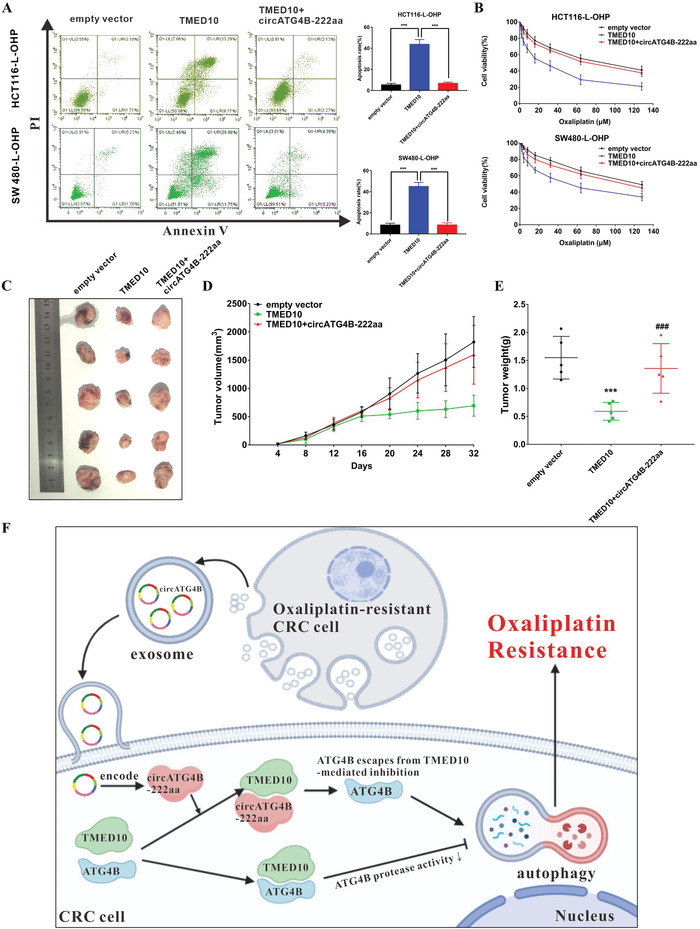
CircATG4B‐222aa reverses chemosensitivity induced by TMED10 in oxaliplatin‐resistant CRC cells. A) CircATG4B‐222aa reversed the increases in apoptosis induced by TMED10 in chemoresistant CRC cells. B) CircATG4B‐222aa reversed the increases in chemosensitivity induced by TMED10 in chemoresistant CRC cells. C) Nude mice xenografts were formed by HCT116‐L‐OHP cells transfected with empty‐vector, TMED10, and TMED10+circATG4B‐222aa, respectively. Then, sixteen days after injection, oxaliplatin was injected intraperitoneally. D) Tumor volumes were monitored during the time course. E) Tumor weights were also measured at the end of this study. F) CircATG4B‐222aa could induce oxaliplatin resistance by interacting with TMED10 and increasing autophagic activity in CRC cells. ***p < 0.001; ***p < 0.001 empty vector versus TMED10 group, ### *p* < 0.001 TMED10 versus TMED10+circATG4B‐222aa group (e).

Subsequently, we assessed the function of the interaction between circATG4B‐222aa and TMED10 in oxaliplatin resistance. First, we constructed a TMED10 overexpression plasmid and found that it upregulated the expression of TMED10 in chemoresistant CRC cells (Figure S7, Supporting Information). As expected, circATG4B‐222aa reversed the increases in apoptosis and chemosensitivity induced by TMED10 in chemoresistant CRC cells (**Figure** **7**A,B). To further investigate the functional links between circATG4B‐222aa and TMED10 in vivo, mice bearing xenografts derived from chemoresistant CRC cells stably transfected with different plasmids were treated with oxaliplatin on the Day 16. There was a significant decline in both of tumor growth and weight in the group implanted with TMED10‐overexpressing cells, while this tumor‐suppressive effect was reversed by overexpressing circATG4B‐222aa (Figure 7C–E). In summary, these results indicated that circATG4B‐222aa could induce oxaliplatin resistance by interacting with TMED10 and increasing autophagic activity (Figure 7F).

## 3 Discussion

Oxaliplatin resistance has historically hindered the treatment effect and led to poor prognosis in CRC patients. However, the biological mechanisms by which circRNAs play a role in chemoresistance in CRC remain unclear. This study indicated that exosomal circATG4B performs a biological function in enhancing chemoresistance. CircATG4B, which was upregulated in oxaliplatin‐resistant CRC cells, could be transferred to sensitive cells via exosomes. A novel protein (circATG4B‐222aa) encoded by circATG4B increased ATG4B activity by competitively binding to TMED10 and in turn induced autophagy, leading to the chemoresistance phenotype. These results shed light on a new target for chemoresistant CRC.

Recently, several studies have indicated that intercellular communication plays an important role in the processes of immunosuppression; cell proliferation, migration and invasion; and chemoresistance.^[^ ^33^, ^34^, ^35^, ^36^ ^]^ Exosomes, an important mechanism for cell signal transduction, can reflect the malignant characteristics of donor cells and transfer specific bioinformation to recipient cells, leading to cancer progression. The bilayer lipid membrane of exosomes protects cellular contents, including miRNAs, circRNAs and proteins, from degradation, permitting their use in intercellular communication. A previous study showed that circRNAs were enriched in exosomes compared to the producer cells.^[^ ^37^ ^]^ Additionally, exosome‐transmitted circRNAs perform biological functions in drug resistance. For example, in hepatocellular carcinoma, cancer cell‐derived exosomal circUHRF1 induces natural killer cell exhaustion and causes resistance to anti‐PD1 therapy.^[^ ^38^ ^]^ Another study reported that exosome‐delivered circRNA (ciRS‐122) promotes glycolysis to induce chemoresistance through the miR‐122‐PKM2 axis in CRC.^[^ ^15^ ^]^ Similarly, in this study, we found that the exosomal circATG4B level was associated with oxaliplatin resistance in CRC. Therefore, stable exosomal circRNAs can be potential biomarkers for CRC in clinical applications.

Generally, recent mechanistic investigations have demonstrated that autophagy processes are involved in the development of chemoresistance.^[^ ^39^ ^]^ Autophagy can play a protective role in cancer cells by eliminating chemotherapy‐damaged organelles, which prevents cancer cells from undergoing programmed cell death. It has been reported that circRNAs regulate autophagy in cancer progression. For example, circCUL2 regulates cisplatin resistance in gastric cancer by modulating autophagy through the miR‐142‐3p/ROCK2 axis.^[^ ^40^ ^]^ ATG4B, a human homologue of ATG4 enzymes, can cleave the precursor LC3 to generate LC3‐I, which is subsequently converted into LC3‐II, leading to the promotion of autophagosome formation.^[^ ^21^, ^22^ ^]^ Given that ATG4B can enhance autophagy, we speculated that ATG4B‐derived circRNA also performs a function in autophagy. In the present study, we evaluated 15 circRNAs derived from ATG4B and found that hsa_circ_0 007159 (circATG4B) was obviously upregulated in oxaliplatin‐resistant CRC tissue. As expected, circATG4B was also shown to regulate autophagy in CRC cells, resulting in oxaliplatin resistance. For clinical analysis, we found that patients with low circATG4B expression but not patients with high circATG4B expression can benefit from oxaliplatin treatment, suggesting that circATG4B might reduce the curative effect of oxaliplatin.

As a novel class of ncRNAs, circRNAs have various biological functions.^[^ ^41^ ^]^ It is well recognized that miRNA sponging is the most commonly reported function of circRNAs. However, our results showed that circATG4B was not associated with AGO2, indicating that circATG4B does not function as a miRNA sponge. Increasing evidence has indicated that circRNAs can actually encode proteins, which has directed research attention toward the protein‐coding function of circRNAs.^[^ ^21^, ^42^ ^]^ With the development of testing approaches, an increasing number of peptides encoded by circRNAs have been found, and their potential biological functions have been identified.^[^ ^43^, ^44^, ^45^, ^46^ ^]^ CircRNA‐encoded proteins usually show functional independence from their host gene products. For example, we previously reported that a protein (circFNDC3B‐218aa) encoded by circFNDC3B could inhibit colon cancer progression though the Snai1/FBP1 pathway, suggesting that circFNDC3B‐218aa performed its biological function independently.^[^ ^47^ ^]^ On the other hand, some products encoded by circRNAs reciprocally affect their host gene. For example, circSHPPH‐146aa protects its host gene SHPPR from degradation^[^ ^48^ ^]^ and circFBXW7‐185aa induces c‐Myc degradation.^[^ ^49^ ^]^ Herein, we found that circATG4B encodes a novel protein termed circATG4B‐222aa. Furthermore, circATG4B‐222aa but not circATG4B itself increased autophagy and subsequently decreased drug sensitivity. According to a previous study, the C‐terminal region of TMED10 is involved in its interaction with ATG4B, while both the N‐terminal and C‐terminal regions of ATG4B might participate in its interaction with TMED10.^[^ ^32^ ^]^ Similarly, we found that full‐length circATG4B‐222aa might also directly bind to the C‐terminal region of TMED10 and probably perform a biological function similar to that of ATG4B. The translated product of circATG4B (circATG4B‐222aa) shares the majority of its amino acid sequence with ATG4B, and they can interact with a similar region of TMED10. Surprisingly, the 24 kDa circATG4B‐222aa protein prevents ATG4B from interacting with TMED10. Because ATG4B is an important factor that frequently performs its biological function in the autophagy process, we inferred that circATG4B‐222aa may act as a protective “decoy” for ATG4B, subsequently preventing the TMED10‐ATG4B interaction and increasing ATG4B protease activity. More importantly, it was this effect that induced CRC chemoresistance. These findings might shed light on the protein‐coding potential of circRNAs during drug resistance progression and provide a precise intervention target for the treatment of oxaliplatin‐resistant CRC.

In summary, we found that the upregulated exosomal circATG4B derived from oxaliplatin‐resistant CRC cells can be transferred to recipient cells, which induce oxaliplatin resistance in the recipient cells. Additionally, circATG4B encodes a novel functional protein (circATG4B‐222aa). CircATG4B‐222aa but not circATG4B itself promoted autophagy and subsequently induced oxaliplatin resistance in CRC cells both in vitro and in vivo. Furthermore, we found that circATG4B‐222aa interacts competitively with TMED10 and functions as a decoy that prevents TMED10 from binding to ATG4B, leading to increased autophagy and inducing drug resistance. Collectively, this study demonstrated a novel molecular mechanism of a circRNA in the acquisition of drug resistance in CRC. The novel circATG4B‐222aa is a vital biomarker that may be a potential therapeutic target for oxaliplatin‐resistant CRC.

## 4 Experimental Section

### Ethical Statement and Tissue Collection

Surgical tissue specimens were collected prior to initiation of oxaliplatin therapy at the Guangdong Provincial People's Hospital between 2015 and 2018. After surgical resection, the specimens were immediately frozen and stored at −80 °C, and their clinical data was collected after each surgery. Patients in oxaliplatin group received at least 6 cycles of oxaliplatin treatment while patients in control group were treated with no chemotherapy or stopped oxaliplatin therapy. The written informed consents were obtained before surgery, and this study was approved by the Ethics Committee of Guangdong Provincial People's Hospital.

### Exosome Purification and Identification

In brief, until the CRC cells reached 70–80% confluence, the medium was replaced with low‐glucose DMEM containing 10% exosome‐free FBS. After incubation for another 48 h, the supernatant was centrifuged at 3200 g for 10 min at 4 °C to pellet the residual cells. Then, samples were ultracentrifuged at 10 000 g for 30 min at 4 °C to discard the cellular debris. The exosomes were harvested by ultracentrifugation twice at 100 000 g for 2 h at 4 °C.

Size distribution of exosomes was determined by NTA using a NanoSight LM10 instrument (Malvern Instru‐ments Ltd, Malvern, UK) equipped with a sCMOS camera. The data was analyzed with the NTA software version 3.1.54. After dilution for ten times with PBS, the samples were assessed. Readings were taken on once per 10 s, at a camera level set to 16 and with manual monitoring of temperature.

### Cell Culture and RNase R Treatment

The human CRC cell lines were acquired from ATCC. The oxaliplatin‐resistant CRC cell lines HCT116/oxaliplatin (HCT116‐L‐OHP), SW480/oxaliplation (SW480‐L‐OHP), HT29/oxaliplation (HT29‐L‐OHP), and DLD1/oxaliplation (DLD1‐L‐OHP) were established via treating the cells with gradual increasing concentrations of oxaliplatin and cultured in regular conditions for the selection of oxaliplatin‐resistant cells. All the cells were cultured with 10% FBS and DMEM at 37 °C with 5% CO_2_. RNase R (Epicentre Technologies, USA) was used to degrade linear mRNA. In brief, RNA extracted from CRC cells was split to two aliquots: one for RNase R digestion and another for control with digestion buffer only. The samples were incubated at 37 °C for 30 min. Then the levels of circATG4B and ATG4B using qRT‐PCR were assessed.

### Total RNA Extraction and Quantitative Real‐time PCR

All primer sequences are shown in Table S1, Supporting Information. The total RNA sample was extracted with TRIzol reagent (Invitrogen, USA) according to manufacturer's protocol. The concentration and purity of all samples were subsequently measured via NanoDrop 2000 (Thermo Scientific, Wilmington, DE, USA). After reverse transcription, cDNA generated from PrimeScript RT Master Mix (TaKaRa, Japan) was amplified by qRT‐PCR using a LightCycler 96 System (Roche, Switzerland). The relative expression was calculated by the 2^−ΔΔCq^ method.

### RNA Fluorescence In Situ Hybridization

RNA fluorescence in situ hybridization (RNA‐FISH) was conducted to visualize the location of circATG4B by a Fluorescent in Situ Hybridization Kit (RiboBio, Guangzhou, China) in accordance with the manufacturer's protocol.

### Cell Viability Assay

Followed by different pretreatments and exposure of oxaliplatin at various doses for 48 h, cells were seeded into 96‐well plates. Cell viability was detected with CCK‐8 (CCK‐8, ImmunoWay Biotechnology Company Plano, TX, USA). The absorbance values were determined using OD450 values. Three assays were repeated for three times at least

### Colony Formation Assay

Cells seeded into 6‐well plates were all treated with different pretreatments (oxaliplatin and/or exosome) for 24 h in complete media, washed with PBS, and cultured in complete media for another 7 days. The visible colonies were counted after staining with crystal violet.

### Western Blotting Analysis

The protein was extracted with RIPA buffer (CWBIO, China). The equal amount of samples electrophoresed by SDS‐PAGE was transferred to PVDF membranes. After incubation with primary antibodies at 4 °C overnight, the PVDF membranes with secondary antibodies at room temperature were incubated for 1 h. The immunoreactive signals detection was performed by Immobilon ECL substrate (Millipore, Germany) and Optimax X‐ray Film Processor (Protec, Germany). The antibodies used in this study are listed in Table S2, Supporting Information.

### Transmission Electron Microscopy Analysis

The morphology of exosomes was visualized using TEM. First, at room temperature, exosomes were incubated in 4% paraformaldehyde (PFA). Then, the samples were washed with PBS, resuspended in PBS, and loaded on the Formvar/carbon‐coat grids for 20 min stationary incubation followed by fixing with glutaraldehyde (2%) for 1 h and treating with aqueous phosphor‐tungstic acid (3%) for negatively staining. Finally, after quickly being submerged in ethanol (50%) and dried, exosomes were observed on a TEM.

Cells with different treatments were fixed in 2.5% glutaraldehyde at 4 °C overnight, and the ultrathin sections (70–80 nm thick) were prepared using an ultramicrotome (RMC MT6000‐XL). Then the samples were stained with uranyl acetate and lead citrate. Finally, the autophagosomes in the CRC cells were observed under a TEM.

### Autophagosome Detection in Cell Lines

LC3 (MAP 1LC3B, Microtubule Associated Protein 1 Light Chain 3 Beta) is a recognized autophagic marker. During the formation of autophagosomes, cytosolic LC3 (LC3‐I) would be transferred to membrane‐bound LC3 (LC3‐II) which is a characteristic for autophagosome formation. P62, another autophagic marker, serves as scaffold for the formation of protein aggregates, and it acts as an autophagy receptor by linking ubiquitin‐tagged protein aggregates to autophagosomes for degradation.^[^ ^50^ ^]^ In this study, immunofluorescent staining was performed to observe the autophagosome formation in CRC in each group following the previous described.^[^ ^51^ ^]^ After treated according to the group design, the cells were washed twice with PBS and fixed with 4% paraformaldehyde (PFA) at room temperature for 30 min followed by the treatment with 0.5% Triton X‐100 for 5 min. Then, 3% bovine serum albumin (BSA) was used to block non‐specific antigen, and, subsequently, the samples were incubated with the primary antibody overnight at 4 °C. After washing with 0.05% Triton X‐100 for three times, the samples were incubated with Alexa Flour 594‐conjugated secondary antibodies in the dark for 1 h at room temperature. Finally, the autophagosome formation in the CRC cells was visualized under a Zeiss 880 confocal microscope (Nikon Instruments, Melville, New York, USA).

### Oligonucleotide Transfection

The level of circATG4B in cells was knockdown by siRNA which was generated by GenePharma (GenePharma Corporation, Shanghai, China). The circATG4B overexpressing plasmid was purchased from IGE Biotechnology (IGE Biotechnology, Guangzhou, China). To further differentiate the role of circATG4B‐222aa from circATG4B, four flag labeled vectors for circATG4B using vector (pCDH‐CMV‐MCS‐EF1‐copGFP‐T2A‐Puro) were constructed. The full design of vectors is described in Table S3, Supporting Information. In brief, different cells were seeded in 6‐well plates and cultured to ≈60% before transfection, and cells with siRNAs or plasmids using a Lipofectamine 3000 transfection kit (Invitrogen, USA) were transfected following the manufacturer's protocol.

### Analysis of Peptide Patterns by LC‐MS/MS

To identify the proteins, LC‐MS/MS was performed according to previously described. After immunoprecipitation with antibodies, proteins were subjected to 12% SDS‐PAGE gel. The corresponding protein bands were excised and chopped into 1 mm^3^ pieces. Peptide mixtures extracted from the chopped gels were detecting sequences using the LC‐MS/MS analysis finally.

### Co‐Immunoprecipitation

The cell lysate was harvested using lysis buffer (Thermo Fisher Scientific) and performed the Co‐IP assay using the Pierce Co‐Immunoprecipitation Kit (Thermo Fisher Scientific). The corresponding antibody was first immobilized for 2 h. Then the resin was washed and incubated with cell lysate overnight. After incubation, the resin was again washed, and protein eluted using elution buffer.

### Animal Experiment

Ethical approval was approved by the Ethics Committee of Guangdong Provincial People's Hospital and Sun Yat‐sen University (Guangzhou, China). Oxaliplatin‐resistant tumors were established in 4‐week‐old BALB/c nude mice with HCT116 cells (5 × 10^6^/200 µL PBS). Sixteen days after injection, 20 mg various exosomes or equivalent PBS was injected intravenously every 2 days, and 8 mg kg^−1^ oxaliplatin was injected intraperitoneally every 4 days. The volumes of tumors were measured and calculated every 4 days. As for further study, to assess the effect of circATG4B‐222aa in vivo, four lentivirus vectors for circATG4B (empty vector, circATG4B, circATG4B‐mut, and circATG4B‐222aa) were transfected stably into cell lines. The mice were injected the above stable transfected cells, respectively. Sixteen days after injection, oxaliplatin was injected intraperitoneally. The sizes of tumors were measured every 4 days and their volumes were calculated. Thirty‐two days later, the mice were sacrificed, and the tumors were prepared for IHC. Finally, the interaction between circATG4B‐222aa and TMED10 was assessed. The mice were injected with chemoresistant CRC cells transfected with different vectors. After sixteen days, the mice were intraperitoneally injected with oxaliplatin.

### Immunohistochemistry Staining

First, tissue slides were dehydrated and rehydrated followed by antigen repairmen with sodium citrate buffer. Subsequently, the sections were blocked with normal goat serum at room temperature and then incubated with primary antibodies at 4° overnight, followed by incubation with the secondary antibody at 37° for 30 min. Finally, the sections were detected under a light microscope (Leica, Germany) after treated with DAB.

### ATG4 Activity Assay (Luciferase Activity Assay)

According to the previous studies, an N‐terminal deleted form of Gaussia luciferase (dNGLUC) was used to analyze the cellular ATG4B activity.^[^ ^52^, ^53^ ^]^ In brief, CRC cells transfected with vectors were transiently transfected with pEAK12‐Actin‐LC3‐dNGLUC. When dNGLUC was fused to the C‐terminus of *β*‐actin, dNGLUC activity was not detected in supernatant. However, when LC3 was cleaved by ATG4B, dNGLUC was increased in supernatant in the protein expressing cells. After 48 h of transfection, the dual luciferase reporter assay system was performed to assess the result.

### Statistical Analysis

The data are showed as mean±standard deviation (SD) or as values. Unpaired two‐tailed Student's *t*‐test and X^2^‐test were used to evaluate the correlation between circATG4B and clinical characteristics. Kaplan–Meier curves and Log‐rank tests were used to analyze the DFS. The univariate and multivariate Cox proportional hazards models were used to analyze the independent prognosis factors. Statistical analyses were performed using Stata (version 13.1, StataCorp, College Station, TX) and GraphPad Prism 7 software (GraphPad Software, San Diego, CA). *p* value < 0.05 was considered statistically significant.

## Conflict of Interest

The authors declare no conflict of interest.

## Author Contributions

Z.P., J.Z.,J.Z., and J.L. contributed equally to this work. Z.P., J.Z., J.Z., and J.L. designed this study. Z.P., J.Z. and J.Z. performed the experiments; Z.L., H.F. and J.L. collected tissue samples and the clinical data; Z.P., J.L. and J.L. analyzed and interpreted the data; Z.P. and J.Z. drafted the manuscript, Z.P. edited the manuscript. Y.L., D.W., and J.W. supervised the study. All the authors read and approved the final manuscript.

## Supporting information

Supporting Information

## Data Availability

The data that support the findings of this study are available in the supplementary material of this article.
