# Supplementary material for: A Novel Protein Encoded by Exosomal CircATG4B Induces Oxaliplatin Resistance in Colorectal Cancer by Promoting Autophagy
Source: Adv Sci (Weinh). 2022 Oct 26;9(35):2204513. doi: 10.1002/advs.202204513 (PMC9762280; doi:10.1002/advs.202204513)
Supplement: Supplementary file 1 — Supporting Information [file ADVS-9-2204513-s001.pdf]

ADVANCED SCIENCE
Open Access

Supporting Information

for *Adv. Sci.*, DOI 10.1002/advs.202204513

A Novel Protein Encoded by Exosomal CircATG4B Induces Oxaliplatin Resistance in Colorectal Cancer by Promoting Autophagy

Zihao Pan, Jun Zheng, Jiebin Zhang, Jiatong Lin, Jianguo Lai, Zejian Lyu, Huolun Feng, Junjiang Wang, Deqing Wu* and Yong Li**

Supplementary Materials for

A novel protein encoded by exosomal circATG4B induces oxaliplatin resistance in colorectal cancer by promoting autophagy

Zihao Pan, Jun Zheng, Jiebin Zhang, Jiatong Lin, Jianguo Lai, Zejian Lyu, Huolun Feng, Junjiang Wang, Deqing Wu, Yong Li

Correspondence to: Yong Li (liyong@gdph.org.cn); Deqing Wu (13902301120@139.com); Junjiang Wang (sywangjunjiang@scut.edu.cn)

Supplementary Table S1. The primer sequences used in this study

Primer name	Primer sequence
Primers for real-time PCR	
CircATG4B	F: CATCGCTGTGATTGGAGGTG<br>R: GGAGCTCCACGTATCGAAGA
liner ATG4B	F: GGTGTGGACAGATGATCTTTGC<br>R: CCAACTCCCATTTGCGCTATC
GAPDH	F: GGAGCGAGATCCCTCCAAAAT<br>R: GGCTGTTGTCATACTTCTCATGG
divergent GAPDH	F: GAAGGTGAAGGTCGAGTC<br>R: GAAGATGGTGATGGGATTTC

Supplementary Table S2 Antibodies used in this study

Antigens	Manufacturers	Applications
ATG4B	ab205718, Abcam	1:1000 for WB<br>1:100 for IHC
CircATG4B-222aa	Generated by absin(China)	1:1000 for WB
LC3B	#83506, Cell Signaling Technology	1:1000 for WB<br>1:200 for IHC
GAPDH	#5174, Cell Signaling Technology	1:2000 for WB
dNGLUC	E8023S, New England BioLabs	1:1000 for WB
HRP-linked anti-rabbit IgG	#7074, Cell Signaling Technology	1:10000 for WB
HRP-linked anti-mouse IgG	#7076, Cell Signaling Technology	1:10000 for WB

Supplementary Table S3. The sequence of vector of circATG4B

1.overxpressing-circATG4B

ATTGGAGGTGGACACAAAGGAAGAGGCAGCCAGACAGCTACTTCAGCGTCCTCAACGCATTCATCGA
CAGGAAGGACAGTTACTACTCCATTCACCAGATAGCGCAAATGGGAGTTGGCGAAGGCAAGTCCATA
GGCCAGTGGTACGGGCCCAACACTGTCGCCCAGGTCCTGAAGAAGCTTGCTGTCTTCGATACGTGGA
GCTCCTTGGCGGTCCACATTGCAATGGACAACACTGTTGTGATGGAGGAAATCAGAAGGTTGTGCAG
GACCAGCGTTCCCTGTGCAGGCGCCACTGCGTTTCCTGCAGATTCCGACCGGCACTGCAACGGATTCC
CTGCCGGAGCTGAGGTCACCAACAGGCCGTCGCCATGGAGACCCCTGGTACTTCTCATTCCCCTGCGC
CTGGGGCTCACGGACATCAACGAGGCCTACGTGGAGACGCTGAAGCACTGCTTCATGATGCCCCAGT
CCCTGGGCGTCATCGGAGGGAAGCCCAACAGCGCCCACTACTTCATCGGCTACGTTGGTGAGGAGCT
CATCTACCTGGACCCCCACACCACGCAGCCAGCCGTGGAGCCCACTGATGGCTGCTTCATCCCGGACG
AGAGCTTCCACTGCCAGCACCCGCCGTGCCGCATGAGCATCGCGGAGCTTGACCCGTCCATCGCTGTG

2. circATG4B-flag

ATTGGAGGTGGACACAAAGGAAGAGGCAGCCAGACAGCTACTTCAGCGTCCTCAACGCATTCATCGA
CAGGAAGGACAGTTACTACTCCATTCACCAGA(3xflag)TAGCGCAAATGGGAGTTGGCGAAGGCAAGT
CCATAGGCCAGTGGTACGGGCCCAACACTGTCGCCCAGGTCCTGAAGAAGCTTGCTGTCTTCGATACG
TGGAGCTCCTTGGCGGTCCACATTGCAATGGACAACACTGTTGTGATGGAGGAAATCAGAAGGTTGTG
CAGGACCAGCGTTCCCTGTGCAGGCGCCACTGCGTTTCCTGCAGATTCCGACCGGCACTGCAACGGAT
TCCCTGCCGGAGCTGAGGTCACCAACAGGCCGTCGCCATGGAGACCCCTGGTACTTCTCATTCCCCTG
CGCCTGGGGCTCACGGACATCAACGAGGCCTACGTGGAGACGCTGAAGCACTGCTTCATGATGCCCC
AGTCCCTGGGCGTCATCGGAGGGAAGCCCAACAGCGCCCACTACTTCATCGGCTACGTTGGTGAGGA
GCTCATCTACCTGGACCCCCACACCACGCAGCCAGCCGTGGAGCCCACTGATGGCTGCTTCATCCCGG
ACGAGAGCTTCCACTGCCAGCACCCGCCGTGCCGCATGAGCATCGCGGAGCTTGACCCGTCCATCGCT
GTG

3. circATG4B-flag(mut)

ATTGGAGGTGGACACAAAGGAAGAGGCAGCCAGACAGCTACTTCAGCGTCCTCAACGCATTCATCGA
CAGGAAGGACAGTTACTACTCCATTCACCAGA(3xflag)TAGCGCAAACGGGAGTTGGCGAAGGCAAGT
CCATAGGCCAGTGGTACGGGCCCAACACTGTCGCCCAGGTCCTGAAGAAGCTTGCTGTCTTCGATACG
TGGAGCTCCTTGGCGGTCCACATTGCAATGGACAACACTGTTGTGATGGAGGAAATCAGAAGGTTGTG
CAGGACCAGCGTTCCCTGTGCAGGCGCCACTGCGTTTCCTGCAGATTCCGACCGGCACTGCAACGGAT
TCCCTGCCGGAGCTGAGGTCACCAACAGGCCGTCGCCATGGAGACCCCTGGTACTTCTCATTCCCCTG
CGCCTGGGGCTCACGGACATCAACGAGGCCTACGTGGAGACGCTGAAGCACTGCTTCATGATGCCCC
AGTCCCTGGGCGTCATCGGAGGGAAGCCCAACAGCGCCCACTACTTCATCGGCTACGTTGGTGAGGA
GCTCATCTACCTGGACCCCCACACCACGCAGCCAGCCGTGGAGCCCACTGATGGCTGCTTCATCCCGG
ACGAGAGCTTCCACTGCCAGCACCCGCCGTGCCGCATGAGCATCGCGGAGCTTGACCCGTCCATCGCT
GTG

4.circATG4B-222aa-flag

ATGGGAGTTGGCGAAGGCAAGTCCATAGGCCAGTGGTACGGGCCCAACACTGTCGCCCAGGTCCTGA
AGAAGCTTGCTGTCTTCGATACGTGGAGCTCCTTGGCGGTCCACATTGCAATGGACAACACTGTTGTG
ATGGAGGAAATCAGAAGGTTGTGCAGGACCAGCGTTCCCTGTGCAGGCGCCACTGCGTTTCCTGCAG
ATTCCGACCGGCACTGCAACGGATTCCCTGCCGGAGCTGAGGTCACCAACAGGCCGTCGCCATGGAG
ACCCCTGGTACTTCTCATTCCCCTGCGCCTGGGGCTCACGGACATCAACGAGGCCTACGTGGAGACGC
TGAAGCACTGCTTCATGATGCCCCAGTCCCTGGGCGTCATCGGAGGGAAGCCCAACAGCGCCCACTA
CTTCATCGGCTACGTTGGTGAGGAGCTCATCTACCTGGACCCCCACACCACGCAGCCAGCCGTGGAGC

CCACTGATGGCTGCTTCATCCCGGACGAGAGCTTCCACTGCCAGCACCCGCCGTGCCGCATGAGCATC
GCGGAGCTTGACCCGTCCATCGCTGTGATTGGAGGTGGACACAAAGGAAGAGGCAGCCAGACAGCTA
CTTCAGCGTCCTCAACGCATTCATCGACAGGAAGGACAGTTACTACTCCATTCACCAGA(3xflag)TAG

Supplementary Table S4. Relationship between circATG4B and clinical characteristics in CRC patients

	Low expression		High expression		P
**TNM stage**					0.833
I and II	15	23.44	14	21.88	
III and IV	49	76.56	50	78.13	
**Tumour location**					0.216
Colon	36	56.25	29	45.31	
Rectum	28	43.75	35	54.69	
**Differentiation**					0.626
Well/Moderate	53	82.81	55	85.94	
Poor	11	17.19	9	14.06	
**Chemotherapy**					1
Control	30	46.88	30	46.88	
Oxaliplatin	34	53.12	34	53.12	
**Age**	61.91±12.08		61.92±12.49		0.994

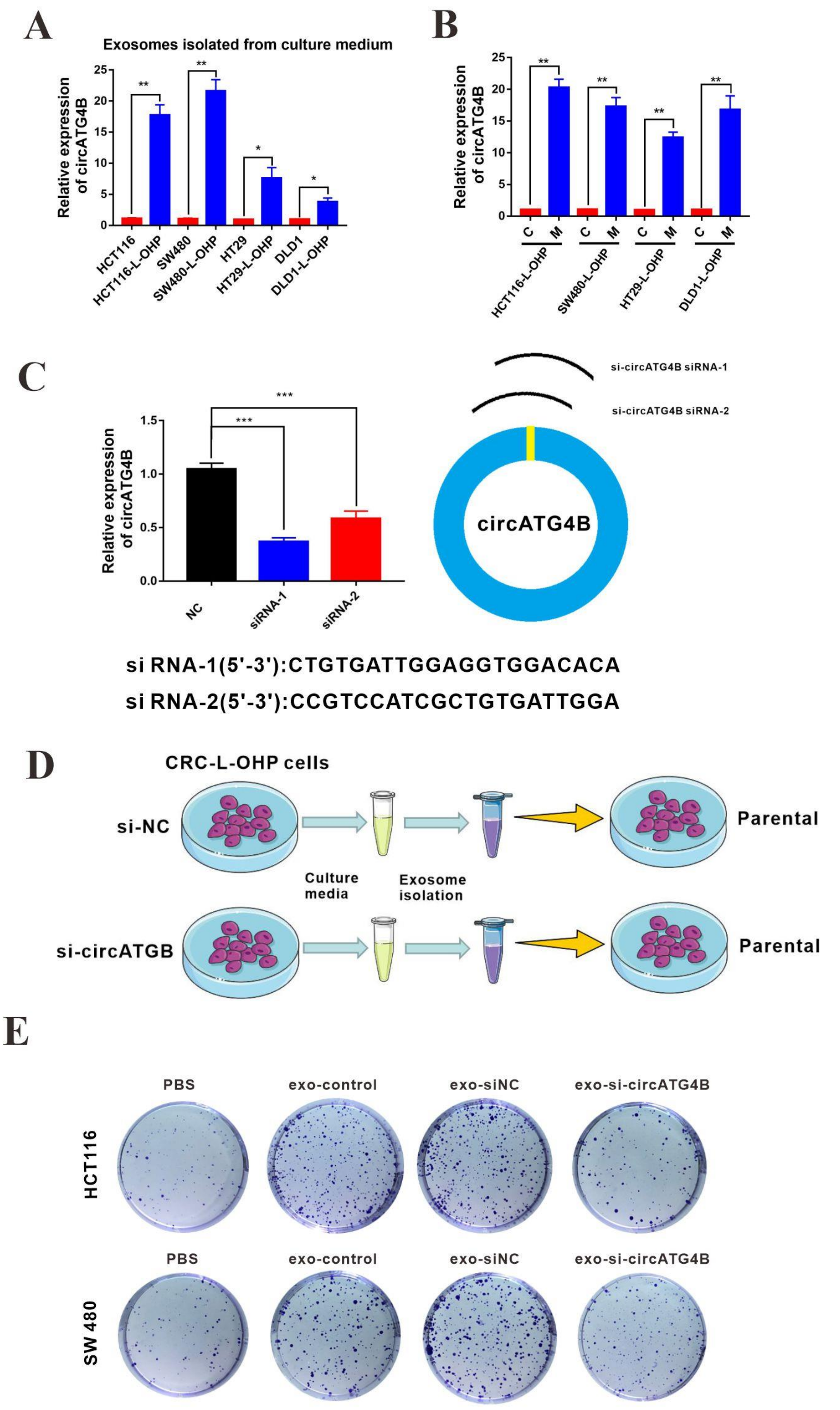

Supplementary Fig.S1 **a** qRT-PCR analysis of exosomal circATG4B isolated from oxaliplatin-resistant and parental cell culture media. **b** qRT-PCR analysis of circATG4B isolated from oxaliplatin-resistant cell culture media (M) and cells (C). **c** Left, qRT-PCR analysis of

circATG4B expression after transfecting HCT116-L-OHP cells with si-NC, si-circATG4B-1, and si-circATG4B-2. Right, schematic representation of where the siRNAs were designed. The siRNA sequences are shown below. **d** Schematic showing that HCT116-L-OHP cells were treated with si-NC or si-circATG4B before the culture media were harvested for exosome isolation and subsequent exosome treatments on HCT116 parental cells. **e** Images of colony formation assay wells after oxaliplatin treatment in CRC cells cultured with exosomes derived from chemoresistant cells.

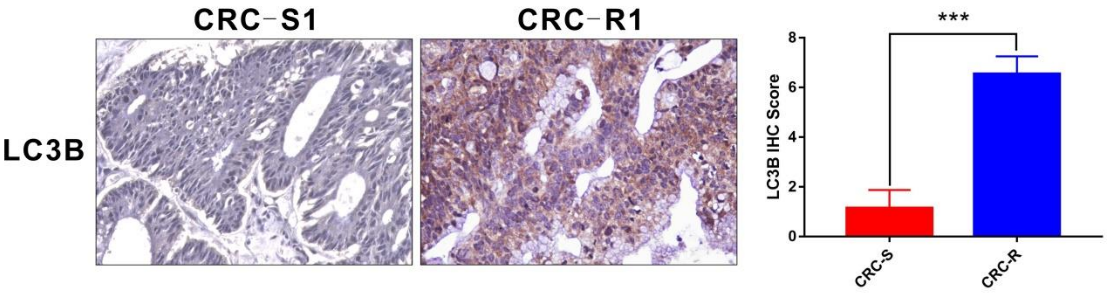

Supplementary Fig. S2 The image of IHC staining of LC3B in clinical chemoresistant and parental CRC samples.

Transcript id	Exon information			
	Spliced_len	Exon Number	Exon Sizes	Exon Offsets
NM_178326, NM_013325	674	6	102, 73, 80, 194, 79, 146	0, 725, 4562, 12099, 13597, 13994

Protein coding potential

IRES Elements	Parameter Index		
	Position (start--end)	R Score	With Pseudoknot (Y/N)
	534--672	1.629623	Y
	580--674	1.538408	Y

Open Reading Frame (ORF)	Start Position	End Position	Protein Length
	108	1r+102	222 aa

MGVGEGKSIG QWYGPNTVAQ VLKKLAVFDT WSSLAVHIAM DNTVVMEEIR RLCRTSVPCA GATAFPADSD RHCNGFPAGA EVTNRPSPWR PLVLLIPLRL GLTDINEAYV ETLKHCFMMP QSLGVIGGKP NSAHYFIGYV GEELIYLDPH TTQPAVEPTD GCFIPDESFH CQHPPCRMSI AELDPSIAVI GGGHKGRGSQ TATSASSTHS STGRTVTTPF TR*

Note:
(1). nr represents n rounds(n<3); (2). * represents a stop codon.

Supplementary Fig. S3 The ORF and IRES site were predicted using circRNADb (http://reprod.njmu.edu.cn/cgi-bin/circrnadb/circRNADb.php).

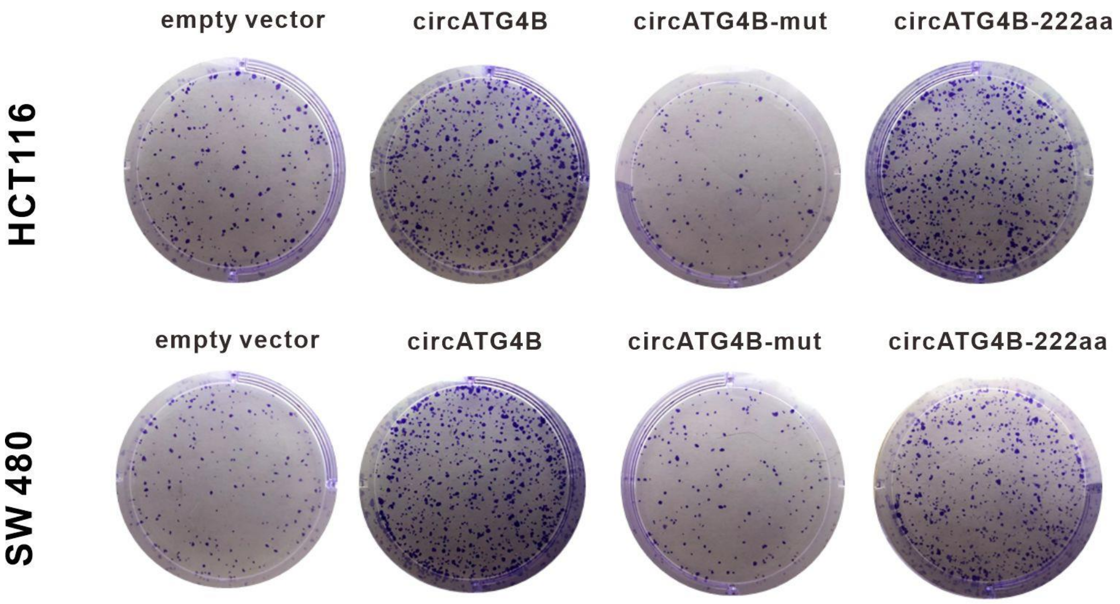

Supplementary Fig. S4 Colony assays show cell viability by oxaliplatin in HCT116 and SW480 transfected with four vectors.

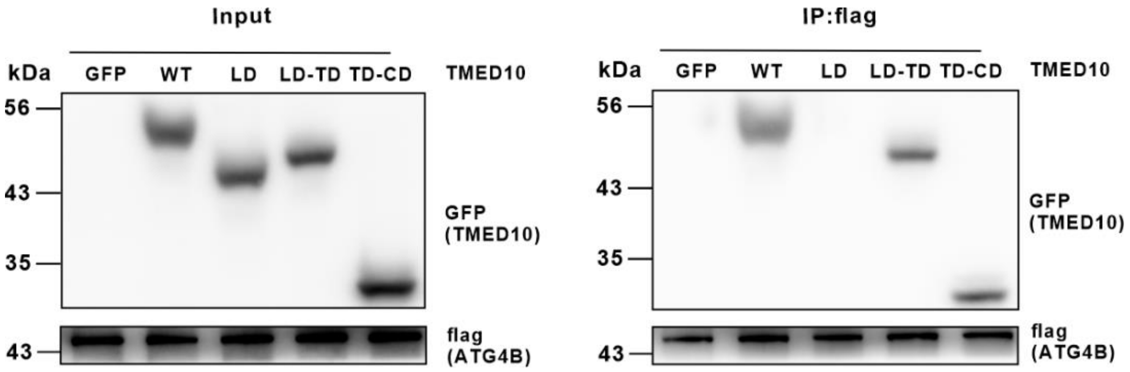

Supplementary Fig. S5 TMED10 directly interacts with ATG4B. 293T cells were transfected with flag-tagged ATG4B and GFP-tagged full-length or TMED10 fragments, followed by IP using anti-flag antibody.

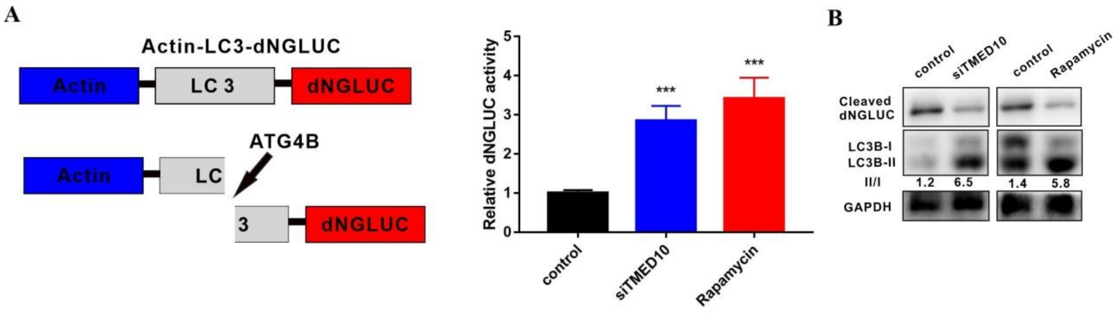

Supplementary Fig. S6 a Downregulation of TMED10 increases autophagy by enhancing the proteolytic activity of ATG4B. Left panel, Schematic diagram of the quantification of ATG4B activity using an assay based on a luciferase-release system. Right panel, HCT116 cells transfected with pEAK12-Actin-LC3-dNGLUC were transfected with control, or siTMED10, or treated with Rapamycin. The supernatants were collected, and the relative luciferase activity was measured. **b** The collected supernatants were analysed by western blotting using an anti-luciferase and anti-LC3 antibody.

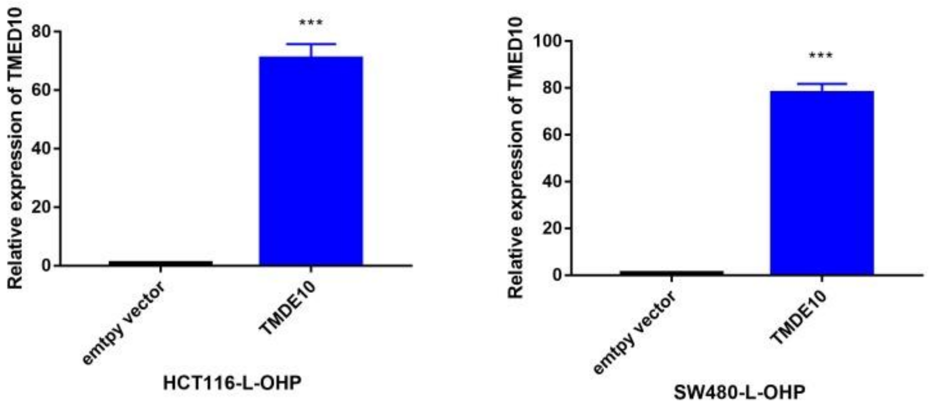

Supplementary Fig. S7 qRT-PCR of TMED10 in CRC chemoresistant cells treated with overexpression plasmid of TMED10
